# An event based topic learning pipeline for neuroimaging literature mining

**DOI:** 10.1186/s40708-020-00121-1

**Published:** 2020-11-23

**Authors:** Lihong Chen, Jianzhuo Yan, Jianhui Chen, Ying Sheng, Zhe Xu, Mufti Mahmud

**Affiliations:** 1 Faculty of Information Technology, Beijing University of Technology, Beijing, 100124 China; 2 Engineering Research Center of Digital Community, Beijing University of Technology, Beijing, 100124 China; 3 Beijing International Collaboration Base on Brain Informatics and Wisdom Services, Beijing University of Technology, Beijing, 100124 China; 4 Beijing Key Laboratory of MRI and Brain Informatics, Beijing University of Technology, Beijing, 100124 China; 5 Department of Computing & Technology, Nottingham Trent University, Nottingham, NG11 8NS UK

**Keywords:** Topic learning, Neuroimaging text mining, Event extraction, Biterm topic model

## Abstract

Neuroimaging text mining extracts knowledge from neuroimaging texts and has received widespread attention. Topic learning is an important research focus of neuroimaging text mining. However, current neuroimaging topic learning researches mainly used traditional probability topic models to extract topics from literature and cannot obtain high-quality neuroimaging topics. The existing topic learning methods also cannot meet the requirements of topic learning oriented to full-text neuroimaging literature. In this paper, three types of neuroimaging research topic events are defined to describe the process and result of neuroimaging researches. An event based topic learning pipeline, called neuroimaging Event-BTM, is proposed to realize topic learning from full-text neuroimaging literature. The experimental results on the PLoS One data set show that the accuracy and completeness of the proposed method are significantly better than the existing main topic learning methods.

## Introduction

Neuroimaging text mining is to extract knowledge from neuroimaging texts [1] and has received widespread attention. Shardlow et al. [2] combined active learning and deep learning to recognize various neuroscience entities for curating research information in computational neuroscience. Riedel et al. [3] recognized various entities related to cognitive experiments based on multiple corpus features and classifiers. Sheng et al. [4] designed the brand new neuroimaging named entity recognition task based on BI provenances and developed the deep learning-based method to recognize these entities for research sharing. However, these studies can only extract valuable information from neuroimaging literature and cannot locate the research focus in literature.

Topic learning can learn the meaningful expression of the text from the document set [5], and obtain information representing document focuses, i.e., topics. Typical topic learning methods are various probabilistic topic models, in which latent Dirichlet assignment (LDA) [6] is the most widely used one. LDA mines the co-occurrence pattern of words to detect the global semantic topic structure and gives document topics in the form of probability distribution. It transforms text information into digital information by using the bag of words. However, this kind of method ignores the word order and the textual structure, and cannot effectively model documents [6]. Various improved LDA models have been developed in order to use the word order and the text structure more effectively for improving topic completeness. Balikas et al. [7] proposed sentence LDA (senLDA) which integrated information of the textual structure and the word dependency into the process of topic modeling. Nguyen et al. [8] proposed LF-LDA and LF-DMM which integrated latent feature models with LDA and DMM by using word embedding. The abilities of topic extraction and classification were greatly improved. The problems of long tail words and low-frequency words were solved effectively. In recent years, many researches began to focus on the combination of deep learning and traditional probability topic models. By using deep neural networks to capture deep semantic information of textual sequences, various shortcomings of traditional probabilistic topic models, which are caused by the shallow feature structure and the probabilistic generation mode, can be overcome to obtain topics with rich and coherent semantics [9]. Dieng et al. [10] proposed the TopicRNN model, which captured the local dependencies between words by using recurrent neural network (RNN) and generated reasonable topics based on global semantic information among potential topics. Yang et al. [11] used candidate topics obtained by LDA to construct feature inputs of the deep neural network and obtained more accurate topics by calculating keywords and the correlation between each pair of feature words.

However, there are still some problems in the above-mentioned topic models, especially poor interpretability and incoherence of topics. Therefore, how to improve topic accuracy is another core issue in current topic learning researches. The above methods combined with deep neural networks can improve semantic coherence of topics by capturing deep semantic representation [12]. Besides this kind of methods, integrating domain knowledge into topic modeling is another important optimization direction of topic modeling [9]. Yao et al. [13] combined LDA with the large-scale probabilistic knowledge base to fuse high-quality prior knowledge in the process of topic modeling. The semantic understanding ability of the model was enhanced to obtain the higher consistency and accuracy of topics. Amplayo et al. [9] proposed a micro-semantic model (MicroASM) that introduced external seed dictionaries into topic modeling for obtaining topics with rich semantics.

Topic learning is also a core research issue of neuroimaging text mining. Neurosynth [14] recognized domain terms based on frequency. Poldrack et al. used LDA to learning topics of neuroscience literature [15]. Alhazmi et al. extracted topic words based on frequency and constructed relations between semantic spaces of topics and brain-activated regions by using correspondence analysis and hierarchical clustering [16]. However, existing researches on neuroimaging topic learning only directly used traditional probability topic models, such as LDA, to extract topics from literature and cannot obtain high-quality neuroimaging topics. Neurosynth topic words include many general words (e.g., using, repeat, asked) and domain general words (e.g., magnetic resonance, brains) [17]. Poldrack et al. had to use concepts in Cognitive Atlas [18] to filter general words without relation to the domain.

Based on the above observations, this paper proposes a topic learning pipeline called neuroimaging Event-BTM, to learn topics from neuroimaging literature. An event-based topic learning task is designed to obtain neuroimaging research topics with rich semantics. Following the trend of fusing deep learning, domain knowledge and probabilistic topic models, a new topic learning method combining deep learning with the biterm topic model (BTM) is proposed. Details are described in the following sections.

## Methods

In this section, we will introduce a new topic learning method oriented to full-text neuroimaging literature, including the definition of neuroimaging research events and the proposed neuroimaging Event-BTM.

### Neuroimaging research events

In the definition of ACE [19], “event” is described as the occurrence of an action or the change of state. Because of rich semantic information, it is naturally more suitable to describe neuroimaging researches than isolated topic words. In order to support event-based topic learning, this paper defines a group of neuroimaging research events by analyzing the process and the result of neuroimaging researches, as well as related information availability in neuroimaging literature. Table 1 gives their definitions.

**Table 1:** Event categories

Topic event	Meta-event	Definition
1. Cognitive response	Activate	The “activate” event happens when the execution of a cognitive task brings about active states in some specific brain regions
	Deactivate	The “deactivate” event happens when the execution of a cognitive task brings about inactive states in some specific brain regions
	Affect	The “affect” event happens when the execution of a cognitive task brings about state changes in some specific brain regions, but whether it is activated or inactivated is unknown
	Co-occur	The “co-occur” event happens when one of the following situations occurs: (1) two brain regions or networks are activated at the same time. (2) Two cognitive tasks are performed at the same time
	Include	The “include” event happens when one of the following situations occurs: (1) an activated brain regions or networks contains another one. (2) A performed cognitive task contains another one
2. Experiment	Design cognitive task	The “design cognitive task” event happens when researchers create a group of cognitive tasks by using design softwares or tools
	Perform cognitive task	The “perform cognitive task” event happens when a subject does a group of experiment tasks during the brain cognitive research
	Acquire data	The “acquire data” event happens when a neuroimaging device records a subject’s physiological and psychological signals during he/she performs cognitive tasks
3. Analysis	Analyze data	The “analyze data” event happens when a researcher adopts some tools or methods to mine neuroimaging data
	Deduce result	The “deduce result” event happens when researchers give a conclusion

As shown in Table 1, neuroimaging research events can be divided into three topic events “Cognitive response”, “Experiment” and “Analysis”, which are used to describe the result, the experimental process and the analytical process of neuroimaging researches, respectively. Each topic event includes several meta-events for the task design of event extraction. According to the definition of ACE, event is composed of an event trigger and several arguments. The event trigger is a word that can trigger the occurrence of an event. It is the most important feature word that determines the event category or subcategory. The argument refers to the participant of an event, which is used to describe the event. By analyzing event mentions in neuroimaging literature, 9 trigger categories and 9 argument categories are identified. All categories are shown in Tables 2 and  3.

**Table 2:** Trigger categories

Category	Definition	Example
Activate	A trigger indicates that the “activate” event occurs	Activation, activity, hyperactivity
Deactivate	A trigger indicates that the “deactivate” event occurs	Deactivation
Include	A trigger indicates that the “include” event occurs	Include, part of, consist of
Affect	A trigger indicates that the “affect” event occurs	Influence, effect, affect
Design cognitive task	A trigger indicates that the “design cognitive task” event occurs	Design, present, record
Perform cognitive task	A trigger indicates that the “perform cognitive task” event occurs	Complete, implement, perform
Acquire data	A trigger indicates that the “acquire data” event occurs	Detectable, examine, assess
Analyze data	A trigger indicates that the “analyze data” event occurs	Performed, use, implement
Deduce result	A trigger indicates that the “deduce result” event occurs	Discuss, analyze, distinguish

**Table 3:** Argument categories

Category	Definition	Example
Gross brain anatomy	The gross brain anatomy is an anatomical region of the cerebral cortex and used to mark the occurrence location of brain response in the brain cognitive research	Auditory cortex
Cognitive function	The cognitive function refers to people’s ability to collect and process information, such as attention, language	Visual perception
Subject	The subject is a participant in the brain cognitive research and recorded for behavioral or brain physiological data	Patient
Medical problem	The medical problem refers to the disease and is used to denote the subject’s abnormal symptom in the brain cognitive research	Hypertension
Sensory stimuli or response	The sensory stimuli or response is used to denote the sensory channel of stimulus presentation in the brain cognitive research	Auditory sense
Experimental task	The experimental task is a task (e.g., questions, games, etc.) which is performed by subjects in the brain cognitive research	Delayed memory task
Experimental measurement	The experimental measurement is a kind of brain testing equipment used in the brain cognitive research	Functional magnetic resonance imaging
Analytical tool and method	The analytical tool and method is a mining algorithm or tool which is used to analyze experimental data in the brain cognitive research	Multivariate analysis
Brain network	The brain network is a kind of brain response which is mined from experimental data in the brain cognitive research	Executive control network

### Event representation

Topic events can be constructed by using meta-events. Therefore, this paper mainly focuses on the extraction and representation of meta-events. These meta-events are usually expressed as a trigger + argument structure [20]. For example, the “deduce result” meta-event can be expressed as follows:

1 $$\begin{aligned}&\text{Event}_{\text{deduce-results}} \\&=\bigl [\text{trigger},<\text{argument}1,\text{role}1>?,<\text{argument}2,\text{role}2>?\bigl ]\\&=\bigl [\{\text{evoke, indicate, reveal}...\},<\{\text{EXPERIMENT TASK}, \\&~~~~~~~~~~\text{COGNITIVE FUNCTION, MEDICAL PROBLEM}\}, \\&~~~~~~~~~~~~\text{research object}>+, \\&~~~~~~~~~~~~<\{\text{FEATURES OF PHYSIOLOGY AND PSYCHOLOGY}\},\\&~~~~~~~~~~~~~~~~\text{biological mechanism}>+\bigl ]\\ \end{aligned}$$

**Fig. 1:**
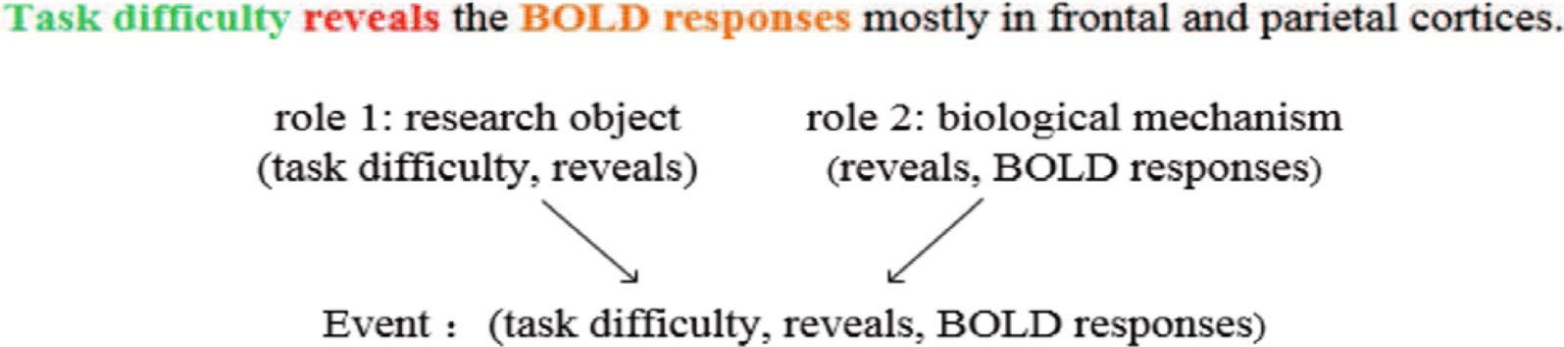
An example of “deduce result” event

It means that a “deduce result” event consists of a trigger, zero or one argument 1, and zero or one argument 2. Various verbs, such as “evoke”, “indicate”, and “reveal”, are possible triggers. Argument 1 belongs to one of three argument categories “experimental task”, “cognitive function” and “medical problem”, and plays the role “research object” during the process of deducing results. Argument 2 belongs to the argument category “signal features of physiology and psychology” and plays the role “biological mechanism”, which is revealed by result deduction. Figure 1 gives an example of “deduce result” event. In this event, there are a trigger “reveals” and two arguments. The argument 1 “task difficulty” is an experimental task and the argument 2 “BOLD responses” is a kind of features of physiology and psychology. Using the distributed vector-based event representation [18], this event can be represented as follows:

2 $$V_E = V_{\text{trigger}} \cdot V_{\text{argument}~1} \cdot V_{\text{argument}~2},$$

where $V_E$, $V_{\text{trigger}}$ and $V_\text{argument}$ are the vector representation of event, trigger and argument, and “$\cdot$” is the point multiplication operation.

### The neuroimaging Event-BTM pipeline

**Fig. 2:**
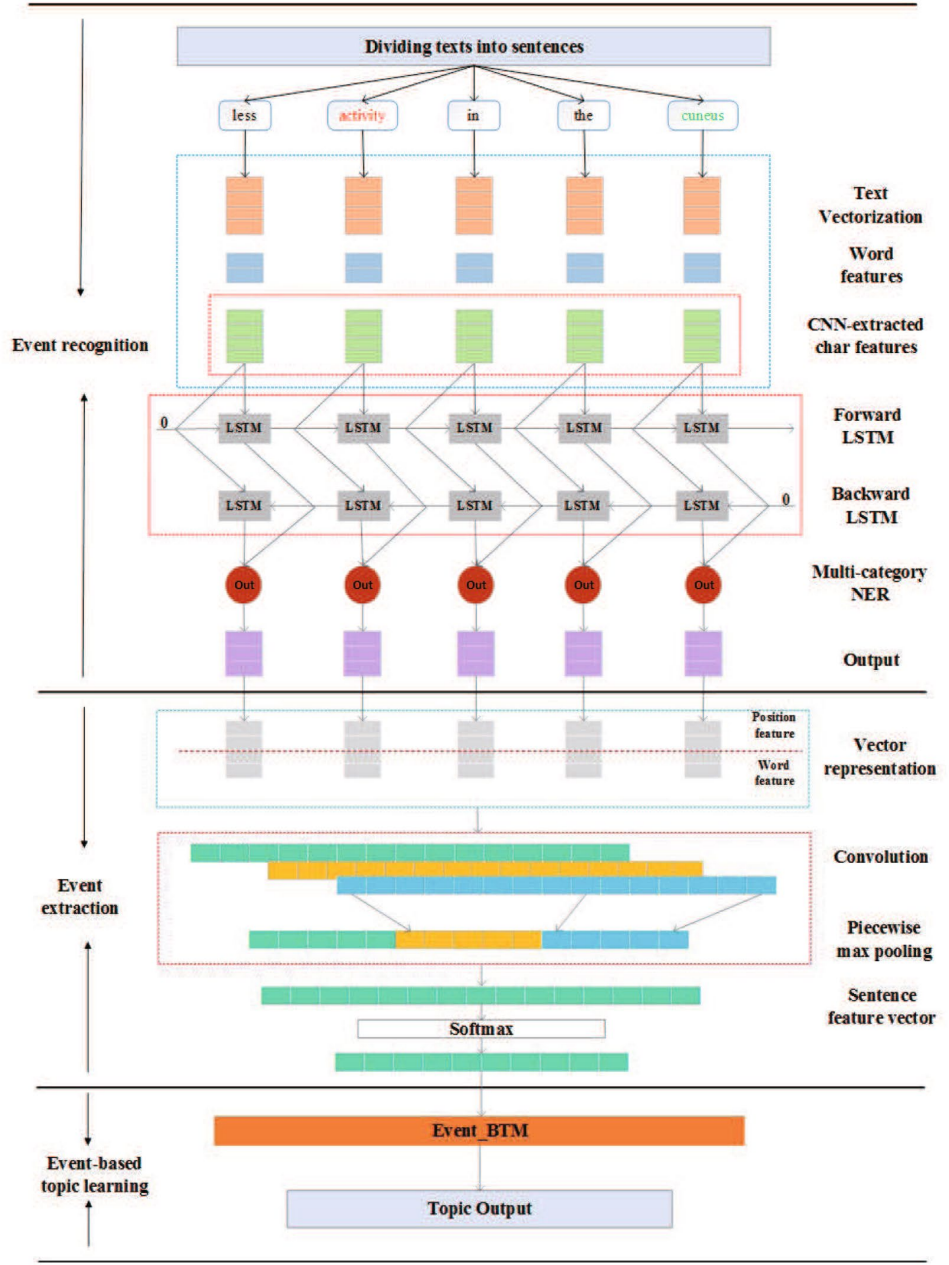
The framework of neuroimaging Event-BTM pipeline

Based on the definition of events in the previous section, we design a topic learning pipeline, called neuroimaging Event-BTM, which combines heterogeneous deep neural networks with BTM. Figure 2 shows the framework of this pipeline. The whole process can be divided into three steps: event recognition, event extraction and event-based topic learning.

#### Event recognition

Event recognition includes trigger recognition, argument recognition and trigger-type recognition. This paper uses BiLSTM-CNN [21] to model textual features for event recognition. As shown in Fig. 2, the whole model consists of two layers: text vectorization and event element recognition.

*Text vectorization* The text vectorization layer encodes each sentence of literature as a textual vector for event element recognition. For each word in the sentence, the following four types of vectors are constructed:

- Word vector: it is to map words into real number vectors to obtain as much semantic and grammatical information as possible [22]. This paper adopts the Glove word vector model which was trained on 6 billion words of Wikipedia and web texts [23]. In order to obtain a rich semantic information of words, this paper retains the stem and affix of each word.
- Case vector: the ten categories of neuroimaging research meta-events and their corresponding arguments involve a large number of domain terms with the capital abbreviation format, such as fMRI (functional magnetic resonance imaging), DMN (default mode network). In order to identify these important upper- and lower-case features, this paper constructs the one-hot case vector which has six dimensions: “numeric”, “allLower”, “allUpper”, “initialUpper”, “mainly_numeric”, “contains_digit” and “other”.
- Terminology dictionary vector: previous study shows that the term dictionary encoding plays an important role in multi-class named entity recognition [23]. Therefore, this paper collects 9 term dictionaries for the 9 argument categories. The one-dimensional terminology dictionary vector is constructed based on these term dictionaries. For each word, the value of its terminology dictionary vector is just the label index of corresponding term in the dictionary.
- Character vector: CNN is used to construct the character vector. Its input is the 25-dimensional character embedding which is generated by looking up the character random table. In this paper, the character random table is initialized with values drawn from an uniform distribution with range [− 0.5, 0.5].

Based on these four types of vectors, a combined word vector can be constructed as follows:

3 $$V_{\text{word}} = [V_w,V_c,V_t,V_{\text{char}} ],$$

where $V_w$, $V_c$, $V_t$ and $V_{\text{char}}$ are the corresponding word vector, case vector, terminology dictionary vector, and character vector.

*Event element recognition* The event element recognition is to decode text vectors for recognizing triggers and arguments. The correct recognition of named entities in sentences depends on the context of the word [24], so it is very important to obtain the context information of the past and future. This paper adopts bidirectional LSTM (BiLSTM) to capture this bidirectional relationship between words. For a sentence, $S = [\text{word}_1, \text{word}_2, \ldots, \text{word}_n]$, the process of feature modeling based on BiLSTM is described as follows:

4 $$h_i = [ \overrightarrow{\text{LSTM}}(v_{\text{word}_i}),\overleftarrow{\text{LSTM}}(v_{\text{word}_i}) ], i=[1,n],$$

where $v_{\text{word}}$ is the combination vector of $\text{word}_{i}$ in the sentence. Based on the output of BiLSTM, log-softmax [25] is used to obtain the log-probability of each trigger or argument type. And then all triggers and arguments are annotated with corresponding category labels.

#### Events extraction

The event extraction is to identify argument roles based on the outputs of event recognition. The lexical-level and sentence-level features are combined to construct the role feature vectors.

The lexical-level role feature is involved with word embedding, annotated triggers or arguments, and the context structure of event mentions. For each role, its lexical-level feature vector is defined as follows:

5 $$V_{lf} = [E_{1t},E_{2t},E_{1tf},E_{1tb},E_{2tf},E_{2tb},r],$$

where $E_{1t}$ is the word vector of the trigger, $E_{2t}$ is the word vector of the argument, $E_{1tf}$ is the word vector of the previous word of the trigger, $E_{1tb}$ is the word vector of the latter word of the trigger, $E_{2tf}$ is the word vector of the previous word of the argument, $E_{2tb}$ is the word vector of the latter word of the argument, and *r* is the index of the role type in the event-role table.

**Fig. 3:**
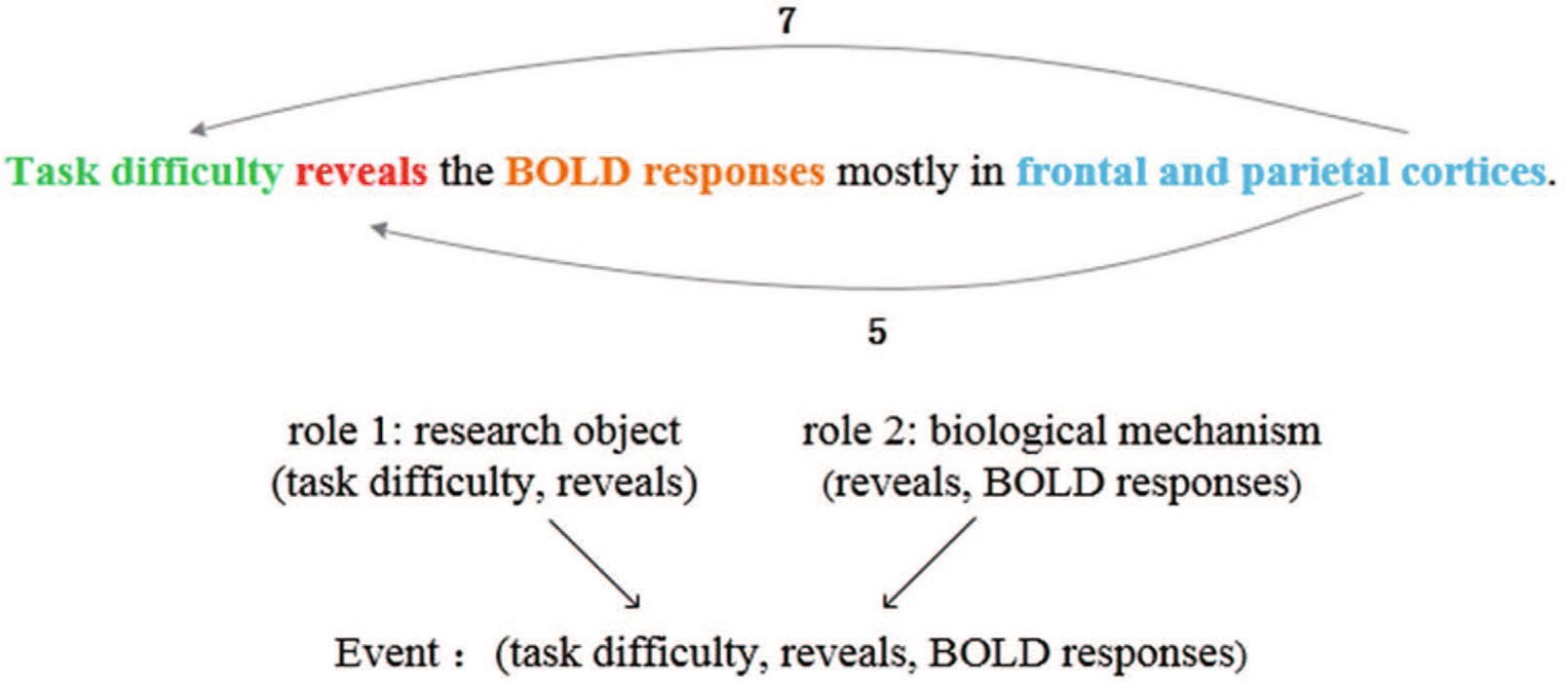
The position embedding

Figure 3 gives an example. For the role “research object”, its $E_{1t}$ is the word vector of “reveals”, $E_{2t}$ is the word vector of “Task difficulty”, $E_{1tb}$ is the word vector of “the”, and *r* is the index of the role type “research object” in the event-role table. Because the argument “Task difficulty” and the trigger “reveals” are next to each other, both $E_{1tf}$ and $E_{2tb}$ are the word vector of NULL whose dimensional values are all zero. $E_{2tf}$ is also the word vector of NULL, because the argument “Task difficulty” is the first word in the sentence.

The sentence-level features are obtained by using CNN [26]. Its input is a group of word representations corresponding to the sentence. For each word, its word representation is as follows:

6 $$v_{\text{wp}} = [v_{\text{wf}},d_{\text{pft}},d_{\text{pfa}}],$$

where $v_{\text{wf}}$ is the word vector of the current word, $d_{\text{pft}}$ is the distance vector between the current word and the trigger, and $d_{\text{pfa}}$ is the distance vector between the current word and the argument. For example, aiming at the role “research object” shown in Fig. 3, $v_{\text{wf}}$ of “frontal and parietal cortices” is its word embedding. Its $d_{\text{pft}}$ is 5 and $d_{\text{pfa}}$ is 7. Based on these word representations, CNN can extract sentence-level global features for predicting the role. The process can be described as follows:

7 $$\begin{aligned} n &= \max(M_1 v_{\text{wp}}),\\ V_{\text{sf}} & = \text{tan}h(W_2 n), \end{aligned}$$

where *W* is the linear transformation matrix of the hidden layer and tan*h* is the activation function.

Linking the lexical-level and sentence-level feature vectors, the role feature vector $v = [V_{\text{lf}},V_{\text{sf}}]$ can be constructed. Based on this role feature vector, the probability of the argument role can be obtained by using the softmax classifier.

#### Event-BTM

**Fig. 4:**
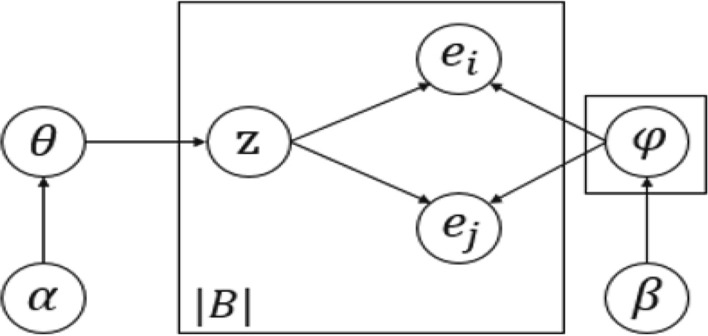
The Event-BTM model

Event-BTM is to learn literature topics from neuroimaging research events which are constructed by integrating the results of event recognition and event extraction. Traditional BTM is to learn all the disordered word pairs in the whole corpus, while Event-BTM directly models all structured event pairs with rich semantics to learn the literature topics. Figure 4 shows the graphical model representation of the model, where the explicit variable *B* is the set of event co-occurrence pairs extracted from the full text of literatures, which is an explicit variable, the topic variable *Z* represents all event biterms, the implicit variables $\varphi$ and $\theta$ are the topic event distribution parameter and the topic distribution parameter, respectively. Referring to [20], Table 4 gives the basic symbols used in Event-BTM and their corresponding explanations. The generative process is outlined as follows:

**Table 4:** Symbols and explanations

Symbols	Explanations
*B*	The set of event co-occurrence pairs in all literatures
*b*	An event pair in the set *B*
*e*	Event
$\alpha$	Prior parameters of *B* topic distribution (hyper-parameters)
$\beta$	Prior parameters of event topic distribution (hyper-parameters)
$\theta$	The topic distribution of *B* (implicit variable)
$\varphi$	Event topic distribution (implicit variable)
*z*	Assignment of the current topic
*M*	Total number of literatures

- for each topic *k*: draw a topic-event distribution: $\varphi \sim \text{Dir}(\beta )$
- sample get topic distribution of the set $B: \theta \sim \text{Dir}(\alpha )$
- for each event biterm *b* in the event biterm set *B*:
  - draw a topic assignment: $z \sim \text{Multi}(\theta )$
  - draw two events: $b(e_i,e_j) \sim \text{Multi}(\varphi )$.

The joint probability of an event biterm $b(e_i,e_j)$ can be written as (the process can be described as follows):

8 $$P(b) = \sum _{z}p(z)p(e_i|z)p(e_j|z)=\sum _{z}\theta _z\varphi _{i|z}\varphi _{j|z}.$$

Then, the likelihood of the set *B* is:

9 $$P(B) = \prod _{(i,j)}\sum _{z}\theta _z\varphi _{i|z}\varphi _{j|z}.$$

The conditional probability of the event biterm *b* is calculated by (8), so the topic distribution and topic-event distribution of the set *B* can be obtained by Gibbs sampling. Then the literature-topic distribution is (for details, refer to the literature [27]):

10 $$P(z|d) = \sum _{b}p(z|b)p(b|d).$$

## Experiments

### Baseline methods

In this paper, LDA, LF-LDA, LF-DMM and MicroASM are selected as baseline methods to verify the effectiveness of the proposed method.

#### The LDA-based method

Traditional LDA is the most classical probability topic model. In this paper, LDA was performed on both the full text and the abstract to learn literature topics, respectively.

#### The LF-LDA-based method and LF-DMM-based method

LF-LDA and LF-DMM integrate context information by using the word vector to improve the word-topic mapping learnt. In this paper, LF-LDA and LF-DMM were performed on both full texts and abstracts for comparing with the proposed neuroimaging Event-BTM.

#### The MicroASM-based method

MicroASM improves the topic quality by using external domain knowledge. In this paper, external domain knowledge of MicroASM, i.e., the seed topic word pairs, was constructed based on the term dictionary “Cognitive Function”. This baseline method was also performed on both full texts and abstracts, respectively.

### Experimental settings

#### Data sources

*Original data* The experimental data set is composed of 126 full texts of literature from the journal PLoS One, which contain any one of “fMRI”, “functional magnetic resonance imaging” and “functional MRI” in abstracts and were published from January 2018 to July 2019. The data set was divided into the training set and the test set according to the ratio of 9:1, and the two sets were further divided into the full-text set and the abstract set.

**Fig. 5:**
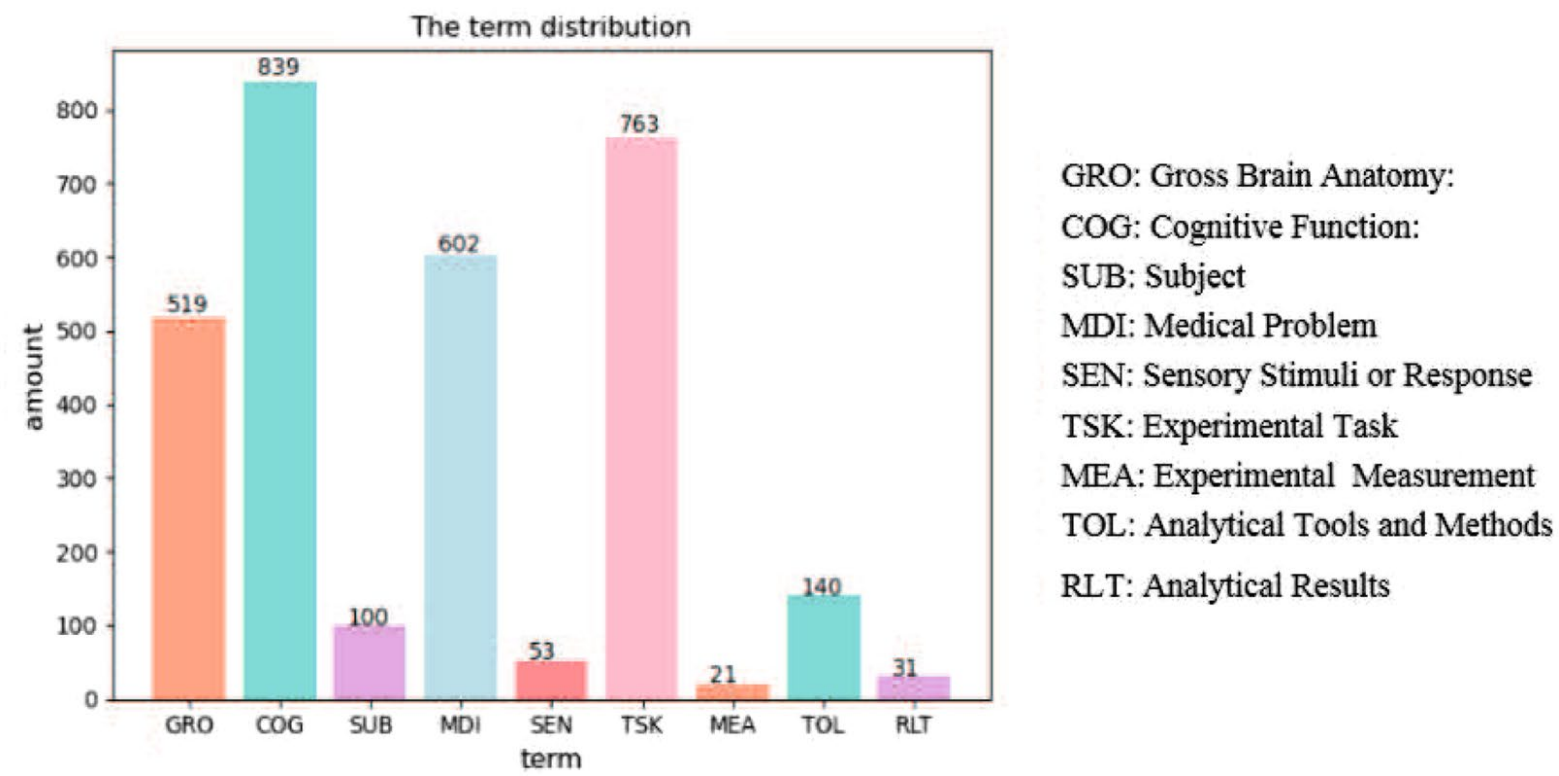
Term distribution

*Term dictionary* Neuroimaging Event-BTM integrates domain knowledge with probability topic models. Existing various domain terms are used to annotate arguments in train data sets by the distant supervision approach. The proportion distribution of 3081 terms is shown in Fig. 5. Their origins are outlined as follows:

- Gross brain anatomy (GRO): 519 brain region terms were collected from the whole brain atlas^1^. They included brain anatomy terms such as “frontal gyri”, “basic ganglia”, “limbic”, and other brain region terms such as Brodmann region.
- Cognitive function (COG): 839 cognitive function terms, such as “attention”, “activation”, “cognitive dissonance”, were collected from “Concepts” terms on cognitive atlas.^2^
- Subject (SUB): 100 crowd-related terms, involved with age, gender, identity, occupation, etc., were collected from the Web.
- Medical problem (MDI): 602 medical problem terms were collected from “diseases”^3^ and “types of cancer” terms on Wikipedia^4^ and Health on the Net.^5^
- Sensory stimuli or response (SEN): 53 perception-related terms, such as “visual”, “auditory” and “emotional”, were collected from the Web.
- Experimental task (TSK): 763 task terms, such as “abstract/concrete task”, “audit diagnostic observation schedule”, were collected from “Tasks” terms on cognitive atlas.^6^
- Experimental measurement (MEA): 21 neuroimaging devices-related terms, such as “MEG (magnetoencephalography)”, “MRI (magnetic resonance imaging)”, “DOI (diffusion optical imaging)”, were collected from the Web.
- Analytical tools and methods (TOL): 140 terms about data-mining algorithms and tools were collected from Baidu Encyclopedia.^7^
- Analytical results (RLT): 31 brain networks-related terms^8^, such as “visual network”, “sensory motor network”, “auditory network”, “cerebellar network”.

#### Experimental annotation

**Fig. 6:**
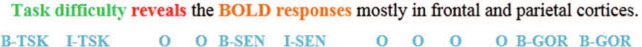
An example of event element annotation

*Event element annotation* In this study, the “BIO” tagging system [28] was used to annotate triggers and arguments. The tagging scheme consists of “–” and the category abbreviation of triggers or arguments. As shown in Fig. 6, “Task difficulty” is an argument belonging to the category “Experimental task”, “BOLD responses” belongs to the category “Gross brain anatomy”, which is a kind of features of physiology and psychology.

**Fig. 7:**
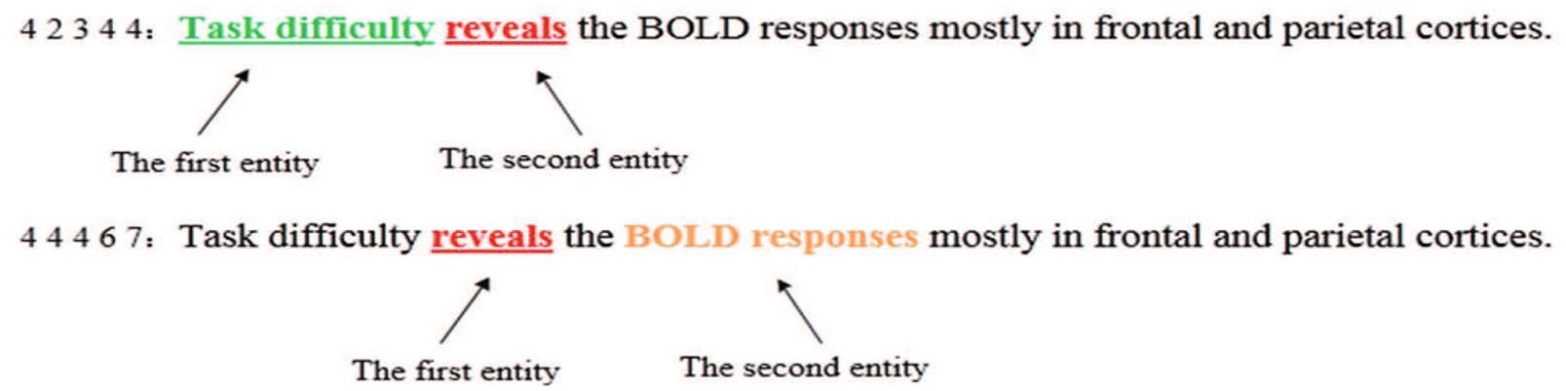
An example of role annotation

*Role annotation* The role annotation consists of five parts: role type, the beginning position of the trigger, the ending position of the trigger, the beginning position of the argument and the ending position of the argument. As shown in Fig. 7 (we just introduce the first sentence), the first “4” represents the relationship between the first entity and the second entity in the sentence, and “2” represents the first entity “Task difficulty”, the second “3” indicates the end position of the first entity, the second “4” indicates the starting position of the second entity “BOLD responses”, and the third “4” indicates the end position of the second entity (because the second entity is a single entity, the start position and end position are the same).

#### Parameter settings

*Experimental parameters* In the experiment, the dimension size of the word vector was set at 100. The epoch was set at 50, the convolution width at 3, the CNN output size at 30, the dimensional number of LSTM hidden layer at 200. We set the number of topics *k* = 2 for all the methods, and set 2 topic words for each topic.

*Evaluation parameters* As stated above, topic accuracy and completeness are two important indexes of topic learning. The former means that the obtained topics should express the document as accurately as possible. The latter means that the obtained topics should summarize the document as comprehensively as possible. This paper chose topic coherence and KL divergence [29, 30] to evaluate the topic model from the two aspects of topic accuracy and completeness, respectively.

Topic coherence is to measure the semantic similarity of topic words in a topic for topic evaluation [29]. It can be calculated as follows:

11 $$\text{coherence}(V) = \text{score}(v_i,v_j,\varepsilon ) = \log\frac{D(v_i,v_j)+\varepsilon }{D(v_j)},$$

where *V* is the set of words in a topic, $\varepsilon$ is the smoothing factor (usually taken directly as 1), $D(v_i,v_j)$ is a function which calculates the number of literature containing words $v_i$ and $v_j$, and $D(v_j)$ is to calculate the number of literature containing $v_j$. Based on the above formulas, the score of topic coherence is the sum of the distributional similarities of topic words in a topic. The higher score of topic coherence indicates the higher topic quality, i.e., better topic accuracy. In this experiment, we calculated topic coherence based on the topic words. This is helpful to explain topics.

KL divergence is the asymmetry measurement of the difference between the probability distributions *P* and *Q*. For topic evaluation, the average KL divergence of topic pairs can be used to measure the difference of topics, i.e., the difference of topic words distribution among different topics. It can be calculated as follows:

12 $$\text{KL}(p||q) = \sum _{x}p(x)\log\frac{p(x)}{q(x)},$$

where *p* is a topic distribution, *p*(*x*) is a topic word in *p*, and *q* is another topic distribution, *q*(*x*) is a topic word in *q*. The higher score of KL divergence indicates the higher topic discrimination, i.e., better topic completeness.

### Experimental results

In the experiment, the proposed neuroimaging Event-BTM and LDA were trained based on the training set, and then four baseline methods were performed on both the abstract test set and full-text test set. The neuroimaging Event-BTM was only performed on the full-text test set because the abstract lacks enough information about neuroimaging research events.

#### Examples of results

**Fig. 8:**
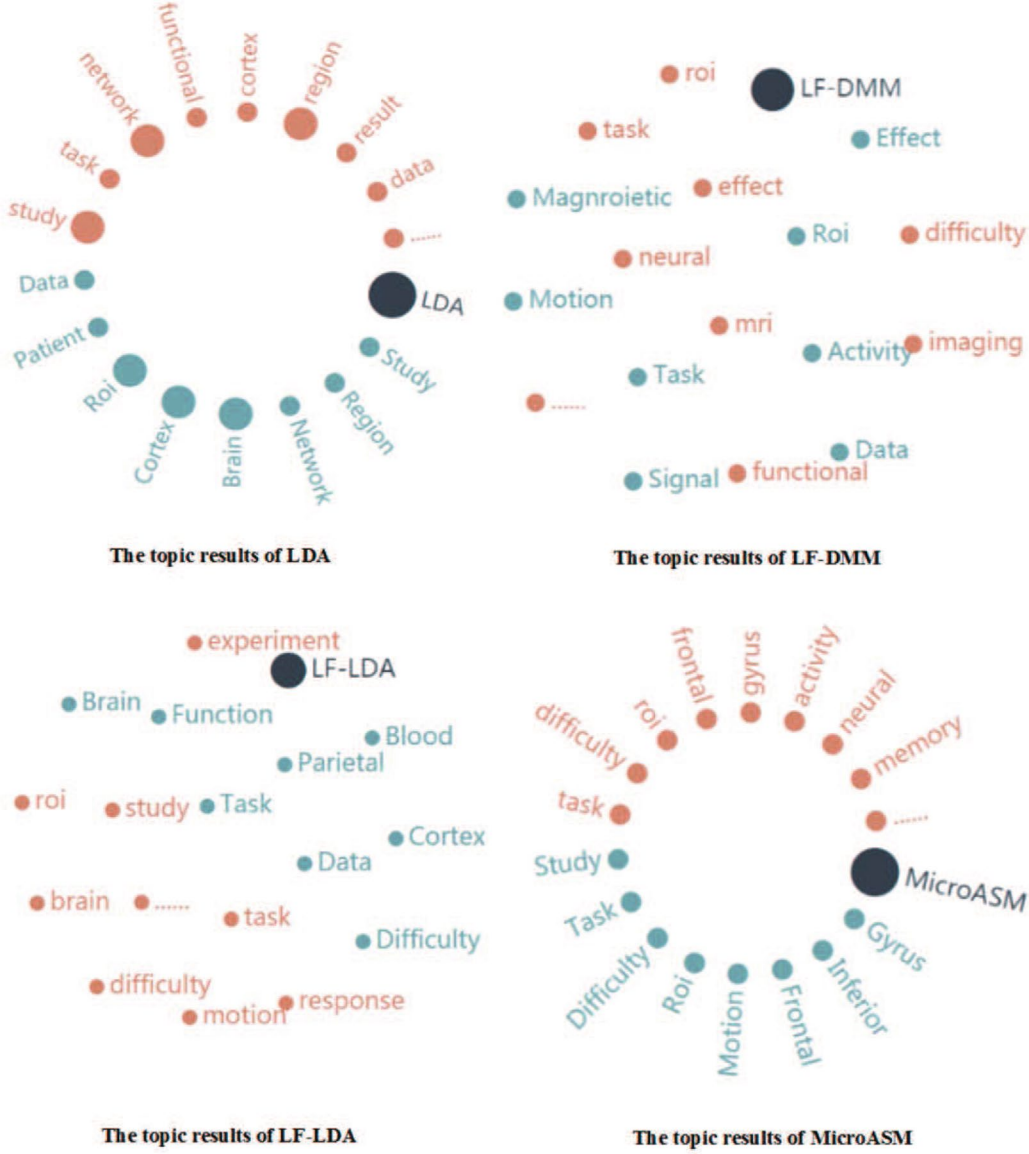
The topic results of baseline methods

*The result of word-based topic learning* Traditional topic learning is based on words. Figure 8 presents the topics extracted from the paper entitled “Effect of task difficulty on blood-oxygen-level-dependent signal: A functional magnetic resonance imaging study in a motion discrimination task” [31], by using the four baseline methods. As shown in this figure, the topic words obtained by LDA, LF-LDA, LF-DMM and MicroASM are scattered and their interpretability is very poor. The reason is that more ambiguous information is brought after semantic association information is segmented. For example, phrases are segmented into multiple words, resulting in unnecessary repetition of words [20]. Therefore, it is necessary to connect several related words to make clear the semantics of the topic. For example, the topic words extracted by LDA include “brain”, “cortex” and “ROI”. Only depending on these isolated domain terms, the research content of literature cannot be understood. Furthermore, the obtained topics often contain polysemic words, such as “study”, “region”, “network”, which are difficult to be understood alone.

**Fig. 9:**
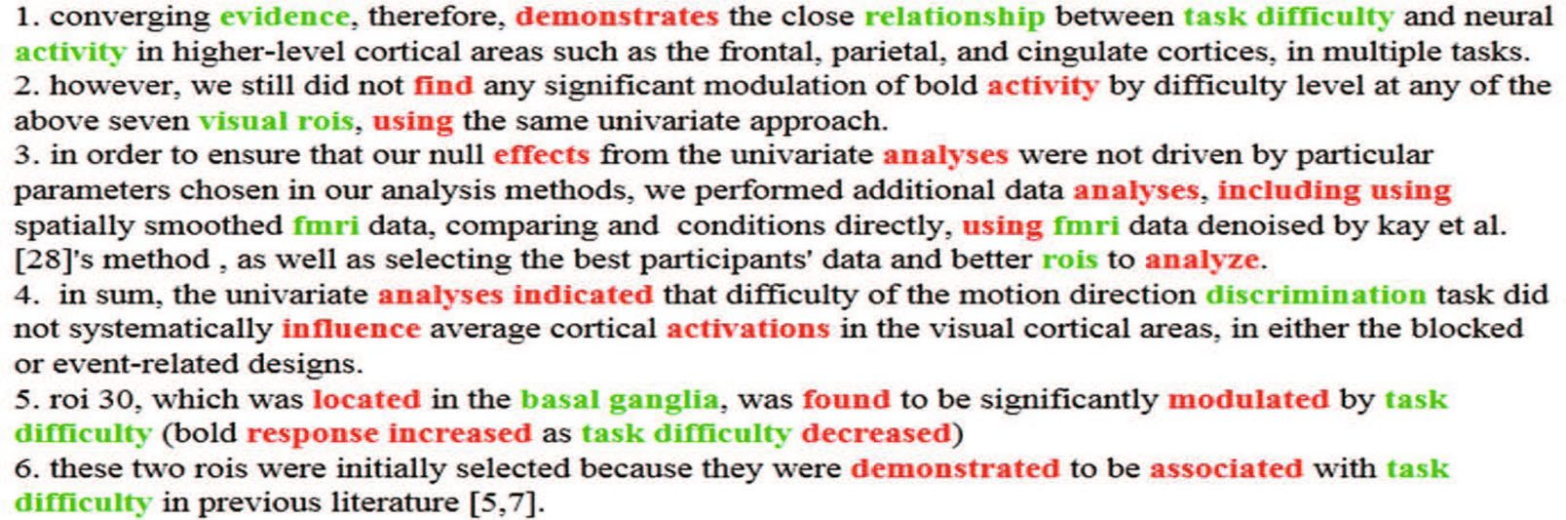
An example of event extraction by using neuroimaging Event-BTM

**Fig. 10:**
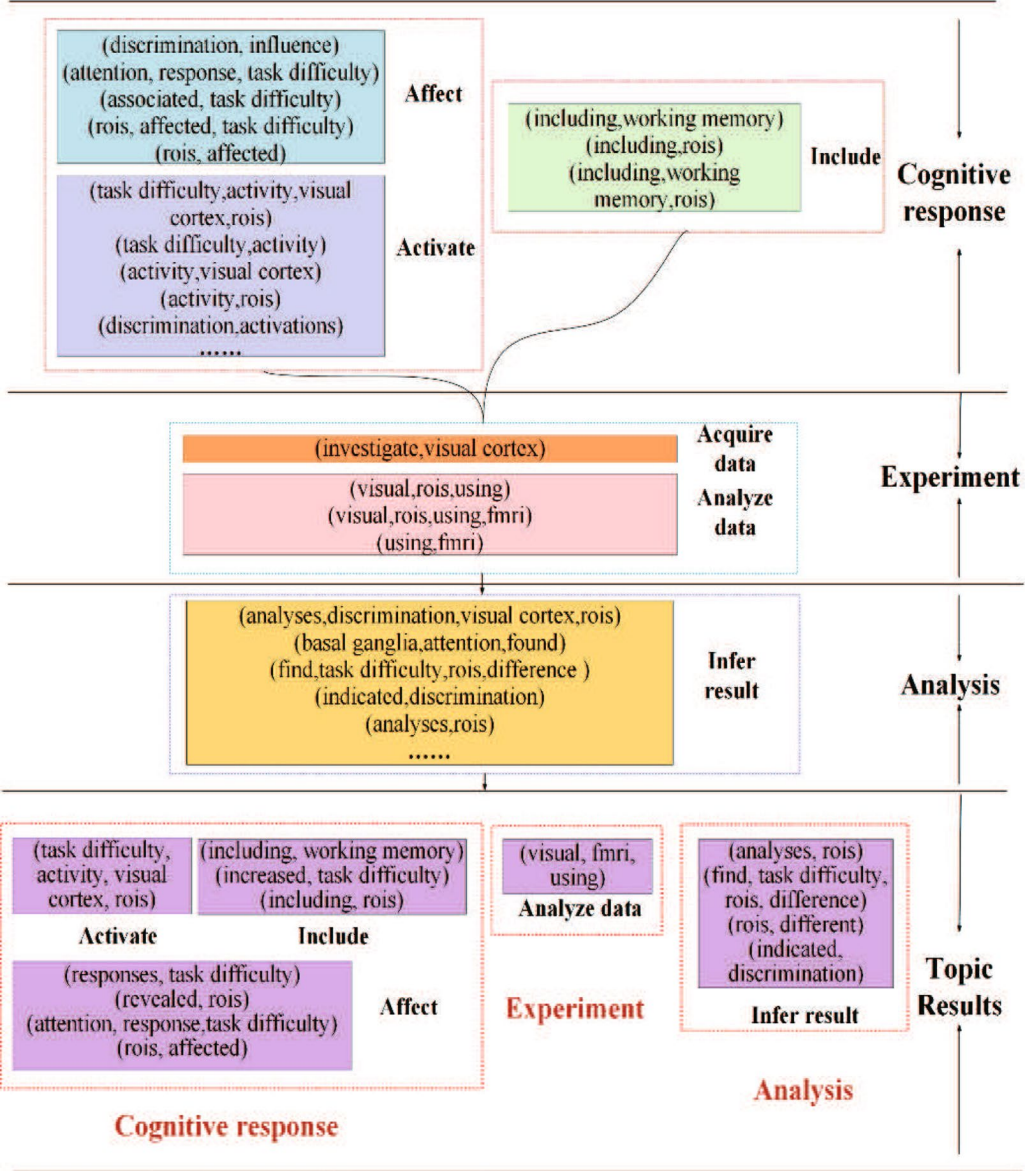
An example of topic learning by using neuroimaging Event-BTM

*The result of neuroimaging Event-BTM* different from the baseline methods, neuroimaging Event-BTM adopts event-based topic learning. Figure 9 shows the part of events extracted from the paper “Effect of task difficulty on blood-oxygen-level-dependent signal: A functional magnetic resonance imaging study in a motion discrimination task” [31]. The red words are trigger words, which can trigger the occurrence of an event, the green words are arguments, which is used to describe the event’s occurrence. All events can be organized into Fig. 10. As shown in this figure, event-based topic representation has better interpretability. The extracted events are involved with the whole research process of the paper, including the experimental task, data collection, parameter analysis, and analysis and result deduction. Finally, topics extracted by Event-BTM, shown in the bottom of the figure, can clearly describe the research focus of this paper. This paper uses the functional magnetic resonance imaging (fMRI) technology to study the affecting of the visual cortex under the different level of difficulty of tasks.

*Evaluation of results* The average topic coherence and average KL divergence of topics that we obtained were calculated. The results are shown in Table 5.

**Table 5:** Experimental results

	Model	Abstract	Full text
Mean topic coherence	LDA	0.87294	0.59431
	LF-DMM	0.07305	0.77734
	LF-LDA	0.33284	0.10402
	MicroASM	0.18297	0.01507
	Neuroimaging Event-BTM		1.55107
Mean KL divergence	LDA	0.00524	0.00022
	LF-DMM	0.00008	-0.00109
	LF-LDA	0.10402	0.04325
	MicroASM	0.01507	0.01450
	Neuroimaging Event-BTM		1.89345

For topic coherence, traditional LDA is better than LF-LDA. This shows that LF-LDA can extract more diversified topic words because it integrates contextual information by using word embedding. DMM assumes that each document is generated by a single topic, but this experiment sets the number of topics $k = 2$. Therefore, topic coherence of LF-DMM is the lowest. Topic coherence of MicroASM is lower than LDA. The reason is that “Cognitive Function” is a core topic and closely related to different categories of topic words in neuroimaging researches. Even if this experiment set “Cognitive Function” as seed topic words, it also cannot effectively constrain the category of co-occurrence topic words for achieving high topic coherence. The proposed neuroimaging Event-BTM achieves the highest topic coherence, which indicates that event-based topic learning can effectively improve topic coherence than traditional word-based topic learning methods, especially under the topic learning task oriented to full texts.

For KL divergence, LF-LDA is better than traditional LDA. This once again confirms that LF-LDA can extract more diversified topic words. The proposed neuroimaging Event-BTM achieves the highest score. This paper introduces domain knowledge into topic learning. Expert knowledge was formalized as three types of neuroimaging research topic events. Formal domain knowledge, i.e., term ontologies or dictionaries, was used to annotate training data by using the distance supervision method. Guiding by domain knowledge, the extracted neuroimaging research events cover the results and processed of neuroimaging researches. Therefore, the topics learned from neuroimaging research events have a higher KL divergence, i.e., topic completeness, and can give more comprehensive literature descriptions than traditional word-based topic learning methods. MicroASM integrates terms belonging to “Cognitive Function” as domain knowledge for topic modeling. Its topic completeness is also improved and achieves the second highest KL divergence.

Furthermore, Table 5 shows that four baseline methods achieve better results on abstracts than on full texts. In recent years, most of international competitions on biomedical text mining, such as BioNLP-ST, have added a large number of full-text corpora to replace the traditional abstract corpora for realizing knowledge learning oriented to the construction of knowledge database. The results in Table 5 show that the full-text mining task bring new challenges. It is difficult to meet the needs only by using the existing technologies on the full texts. The neuroimaging Event-BTM achieved the best results on the full-text topic learning. This effectively reflects the value of our method.

## Conclusion

Currently, neuroimaging topic learning researches mainly adopt typical probability topic models, such as LDA, to extract topics from literature and cannot obtain high-quality neuroimaging topics. Oriented to the full-text topic learning task, existing topic learning methods also cannot effectively meet the requirements of topic learning from full-text neuroimaging literature. For solving this problem, this paper proposes a neuroimaging topic learning pipeline, called neuroimaging Event-BTM, which takes events as the basic unit and extracts topics from neuroimaging full-text literature by combining the deep learning neural network with the topic probability model. These are the following three main contributions:

- By analyzing the process of neuroimaging research and the information availability of neuroimaging literature, three types of neuroimaging research topic events were identified. Based on them, an event-based topic learning task is designed to obtain rich semantic neuroimaging research topics for improving the interpretability and accuracy of topics.
- By fusing deep learning and domain knowledge with probability topic models, a new topic learning method is proposed to realize event-based topic learning oriented to full-text neuroimaging literature.
- Aiming at the two core indexes of topic learning, topic coherence and KL divergence were chosen as evaluation parameters. A group of experiments are completed based on actual data to compare the proposed method with four main topic learning methods.

Experimental results on actual data sets show that neuroimaging Event-BTM can significantly improve topic accuracy and completeness for neuroimaging literature mining.

## Data Availability

Not applicable.

## Footnotes

1 http://www.med.harvard.edu/aanlib/

2 http://www.cognitiveatlas.org/

3 https://en.wikipedia.org/wiki/Listsofdiseases/

4 https://en.wikipedia.org/wiki/Listofcancertypes/

5 http://www.hon.ch/HONselect/RareDiseases/index.html/

6 http://www.cognitiveatlas.org/tasks/a/

7 https://baike.baidu.com/

8 http://www.mamicode.com/info-detail-2224948.htm
